# Extended Latanoprost Release from Commercial Contact Lenses: *In Vitro* Studies Using Corneal Models

**DOI:** 10.1371/journal.pone.0106653

**Published:** 2014-09-10

**Authors:** Saman Mohammadi, Lyndon Jones, Maud Gorbet

**Affiliations:** 1 Systems Design Engineering, University of Waterloo, Waterloo, Ontario, Canada; 2 School of Optometry and Vision Science, University of Waterloo, Waterloo, Ontario, Canada; 3 Centre for Contact Lens Research, University of Waterloo, Waterloo, Ontario, Canada; University of Michigan, United States of America

## Abstract

In this study, we compared, for the first time, the release of a 432 kDa prostaglandin 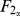 analogue drug, Latanoprost, from commercially available contact lenses using *in vitro* models with corneal epithelial cells. Conventional polyHEMA-based and silicone hydrogel soft contact lenses were soaked in drug solution (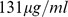 solution in phosphate buffered saline). The drug release from the contact lens material and its diffusion through three *in vitro* models was studied. The three *in vitro* models consisted of a polyethylene terephthalate (PET) membrane without corneal epithelial cells, a PET membrane with a monolayer of human corneal epithelial cells (HCEC), and a PET membrane with stratified HCEC. In the cell-based *in vitro* corneal epithelium models, a zero order release was obtained with the silicone hydrogel materials (linear for the duration of the experiment) whereby, after 48 hours, between 4 to 6 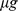 of latanoprost (an amount well within the range of the prescribed daily dose for glaucoma patients) was released. In the absence of cells, a significantly lower amount of drug, between 0.3 to 0.5 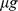, was released, (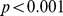). The difference observed in release from the hydrogel lens materials in the presence and absence of cells emphasizes the importance of using an *in vitro* corneal model that is more representative of the physiological conditions in the eye to more adequately characterize ophthalmic drug delivery materials. Our results demonstrate how *in vitro* models with corneal epithelial cells may allow better prediction of *in vivo* release. It also highlights the potential of drug-soaked silicone hydrogel contact lens materials for drug delivery purposes.

## Introduction

Ocular drug delivery is either intended to target the ocular surface to manage superficial conditions such as dry eye, microbial keratitis and conjunctivitis, or to treat intraocular disorders such as glaucoma, and age-related macular degeneration. Eye-drops are still the most common drug delivery method, comprising 90% of ophthalmic medications, followed by ointments and gels [1]. Eye-drop medications are applied topically to the eye in the form of either a solution or suspension in water [2]. The aqueous eye-drop is rapidly diluted in the tear film and most of it is drained through the lacrimal system, therefore, requiring frequent applications [3].

Studies show that only about 1 to 5% of the applied dose penetrates the cornea [4] and that due to the relatively fast turnover rate of the aqueous layer of the tear film, the residence time of hydrophilic medications is around 2 to 5 minutes [5]. The relatively slow turnover rate of the tear film lipid layer results in their increased residence time for lipophilic drugs, which reside in this layer, and consequently results in an increased uptake into the eye. The purpose of topical ophthalmic drug delivery devices is to deliver an adequate amount of medication to the anterior segment of the eye, with accurate targeted dosing at a sustained and controlled rate to increase bioavailability of the drug. Several commercial ocular delivery devices are currently available, including surface-located inserts [6], degradable or non-degradable implants [7], and *in situ* forming gels [8]. Despite almost 50 years of research being conducted on the potential use of soft contact lenses to deliver topical ophthalmic drugs [9], no drug delivery contact lens has yet been commercialized [10].

It is accepted that simple “soaking” of a contact lens in a topical drug solution may be insufficient for adequate elution on the ocular surface; therefore, it is considered to have a low potential for success [11], [12]. Thus a variety of research efforts are attempting to increase the drug uptake and/or release rates. These have included prolonged (up to 2 weeks) soaking [13], soaking the lenses in super-critical drug solutions [14], soaking the dehydrated contact lenses in drug solutions [15], and using vitamin E as a barrier to decrease diffusion of the drugs [16]. However, these efforts have resulted in minimal to no effect on the elution time and release kinetics [11]. It has been documented that the hydrophobic interactions of the active agents (i.e., drugs or other compounds) with the contact lens material is the primary governing factor in the adsorption and subsequent release of these compounds [17].

For the most part, drug release has been studied in a fixed volume of deionized water (DI), Phosphate Buffered Saline (PBS) or artificial tear solutions [11]. In these studies, the drug-eluting contact lens material is placed in a vial with a fixed volume of the release solution, and samples are collected from the solution at various time points. In fixed volume release studies, parameters such as the release medium and its volume, as well as mixing condition, are critically important [11]. The amount of released drug and the elution time have been shown to be consistently smaller when tested using the *in vitro* fixed volume model compared to *in vivo* experiments [12], [14], [18]–[23]. In the fixed volume conditions, the drug release mechanism is governed by diffusion, where concentration gradients generate the driving force and the ratio of the concentration between the contact lens and the medium is dictated by the partition ratio. The fixed volume environment does not represent the ocular environment, where there is a limited amount of tear liquid with a relatively fast tear turnover. The composition of the release medium also plays an important role in release studies. While a contact lens material may present optimal release in DI-water, their performance might be reduced dramatically in the presence of ions or surfactants [24]. In the field of contact lens drug delivery, the inadequacy of current release models has limited progress, primarily by giving rise to a false estimates of the kinetics of release, where the reported behavior cannot be recreated in the physiologic environment. Recently, Byrne et al. introduced a microfluidic device with the purpose of generating physiological flow rates to study the release rate through ophthalmic materials, thus generating a more representative release environment [25]. This microfluidic device mimics tear flow rate and the limited tear volume in the eye.

Considering that the primary drug permeation route to the front of the eye is through the transcorneal pathways, it is also important to consider the role of the cornea in drug release studies [26]. The lipid bilayer cell membrane retards the permeation of hydrophilic compounds. Through expressing certain transporters as well as certain enzymes present in the epithelial cells, the cornea is involved in metabolism and transportation of prodrugs and their active metabolized form [27]–[31]. The corneal epithelium is considered to be the rate-limiting factor in the transcorneal permeation of most ophthalmic drugs [32], [33], especially for hydrophilic drugs [34], [35]. Thus, using an *in vitro* corneal epithelial model will allow replication of the relevant factors of the *in vivo* environment. Human corneal *in vitro* models offer a cost effective and more standardizable substitutes [36] for animal studies while allowing a higher throughput testing of biomaterial interaction and drug permeation [37]. Reconstructed corneal equivalents as well as cell culture models of the corneal epithelium have been successfully used to study ocular toxicity and permeability [37]–[41].

Pharmacokinetics of most prostaglandin 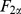 analogues has been extensively studied *in vivo* [4], [42]. The contribution of the enzyme and transport activities such as the esterase activity of the corneal epithelium has been utilized in the design of ophthalmic prodrugs [43]. The lipophilicity, as a result of esterification or amidification of 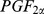 analogues, facilitates the penetration through the cornea. Prostaglandin analogues metabolism into the hydrophilic acid forms inside the epithelial cells allows permeation through the stroma [44] and thus, increases the bioavailability of the active substance in the interior of the eye [45]. We therefore hypothesize that the presence of corneal epithelial cells may have an impact when assessing drug-delivery materials *in vitro*. The objective of this study was to investigate the release of Latanoprost by commercially available contact lenses using *in vitro* models containing corneal epithelial cells.

## Materials and Methods

### Preparation of Drug Doping Solutions

The lens doping solution was prepared by dissolving latanoprost and latanoprost free-acid (solution in methyl acetate, Cayman Chemical, Ann Arbor, MI) in PBS (Lonza, Walkersville, MD). The concentration of the stock drug solution was 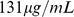.

### Preparation of Contact Lenses

Four commercially available contact lens materials, galyfilcon A, senofilcon A, omafilcon A, and balafilcon A were used. The properties of the four lens types are presented in table 1. All lenses had a back vertex power of -3.00 diopter. Lenses were incubated for 24 hours in PBS (Lonza, Allendale, New Jersey) to remove any remnants of their packaging solutions, before incubation in 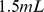 of the drug solution for 24 hours.

**Table 1:** Properties of the Contact Lens Hydrogel Materials [47].

**Commercial name**	Acuvue Advance	Acuvue Oasys	ProClear	PureVision
**(US adopted name)**	Galyfilcon A	Senofilcon A	Omafilcon A	Balafilcon A
**Manufacturer**	Johnson & Johnson	Johnson & Johnson	Coopervision	Bausch & Lomb
**Water content**	47	38	60	36
**Principal Monomer**	mPDMS + DMA + HEMA+ siloxane macromer+ EGDMA + PVP	mPDMS + DMA + HEMA + siloxane macromer + TEGDMA + PVP	HEMA + PC	NVP + TPVC + NVA + PBVC
**FDA group** *	V(I)	V(I)	II	V(III)
	Low water	Low water	High water	Low water
	Non-ionic	Non-ionic	Non-ionic	Ionic

*FDA (Food and Drug Administration) categorizes all silicone hydrogel contact lenses as group V, however it is more practical to use groups for conventional hydrogels to better understand their material properties. HEMA, Hydroxyethyl Methacrylate; PC, Phosphotidylcholine; NVP, N-Vinylpyrrolidone; TPVC, Tris(trimethylsiloxysilyl) Propyvinyl Carbamate; NVA, N-Vinyl Aminobutyric Acid; PBVC, Poly(dimethysiloxy)di (silylbutanol) Bis(Vinyl Carbamate); mPDMS, monofunctional Polydimethylsiloxane; DMA, N, N-Dimethylacrylamide; EGDMA, Ethyleneglycol Dimethacrylate; PVP, Polyvinyl Pyrrolidone; TEGDMA, Tetra-Ethyleneglycol Dimethacrylate

### *In Vitro* Cell Culture

HPV-immortalized human corneal epithelial cells, a generous gift from Dr. May Griffith (Integrative Regenerative Medicine (IGEN) Centre, Linköping University, Sweden) [38] were cultured in keratinocyte serum free medium (KSFM) supplemented with bovine pituitary extract, recombinant epidermal growth factor, and penicillin/streptomycin (Pen/Strep) (ScienCell, Carlsbad, California, USA) at 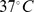 and 5% carbon dioxide (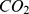). Fresh medium was added every other day and cells were grown to 90% confluency in tissue culture treated flasks. Adherent cells were removed using TryplExpress (Life Technologies, Burlington, Ontario, Canada) dissociation solution. Cells were routinely observed for any morphological changes and were used before their eleventh passage.

### *In Vitro* Drug Release Models

Three *in vitro* models were used to assess drug release from commercially available contact lenses in-cluding diffusion through a) a Polyethylene Terephthalate (PET) membrane (Millicell PET membrane with a 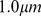 pore size, also referred to as culture inserts, Millipore, MA, USA) with no-cells; b) a PET membrane with a monolayer of human corneal epithelial cells (HCECs) and c) a PET membrane with a multilayer of HCECs (stratified culture). For the two latter models, the PET membranes were seeded with 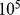 cells. The corneal epithelium models were fed with KSFM on each of the basal and apical sides of the cells layer for five days, with medium being exchanged every other day. After five days, for the multilayer models, cell differentiation was induced by exposing the monolayer to an air-liquid interface. Cells were fed only on the basal side with 2% fetal bovine serum (FBS, Invitrogen, Burlington, ON, Canada) in 1∶1 Dulbeccos minimum essential medium (DMEM, Invitrogen) in Hams F12 nutrient medium (DMEM/F12, Invitrogen); the medium was exchanged daily [41]. The cells grew under these conditions for seven days and were then ready for experimentation.

### Measuring Drug Concentrations

Aliquots of 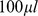 (10% of the total volume of the medium present in the bottom) were taken from the bottom of the *in vitro* models and replaced by fresh culture medium. For the latanaprost experiments, samples were taken at 1, 3, 6, 12, 18, 24 and 48 hours. For latanoprost free-acid experiments, samples were collected at 1, 3, 6, and 24 hours.

Collected samples were analyzed by an enzyme immuno-assay (EIA) for latanoprost (Cayman Chemical, Ann Arbor, MI, USA). Following the EIA kit instructions, each collected sample was analyzed in duplicate and at two different dilutions. To determine the uptake amount by the contact lenses, samples were also aliquoted from the original drug stock solution as well as the remaining drug solutions after soaking the lenses.

The release results represent the concentration of the drug on the other side of the PET membrane, meaning that the drug has been released from the contact lens material on top of the membrane, then diffused through the cells, if present, and the culture membrane. Note that the EIA kit does not distinguish between the free-acid form and ester form of the drug.

### Drug Concentration Calculations

As mentioned above, to measure the amount of released drug, samples were taken from the bottom of the wells and replaced by fresh solution at each time point. Refreshing a fraction of the medium in the bottom at each time point affects measurements. Therefore, it is necessary to account for the dilution effect and adjust the measured concentrations to provide an accurate measure of the concentration without the dilution effect.

Assuming the fraction of total volume of medium in the bottom which is being aliquoted is “
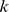
”, the mass balance principle can be used to estimate for the actual concentration at each time point.
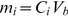
(1)

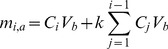
(2)


In Eq.(1), 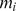 refers to the amount of drug at *i-th* time point (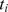), 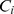 refers to the measured concentration at time 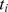, and 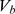 is the volume of the liquid in the bottom. An estimate of the actual amount of drug diffused through the insert adjusted for the dilution effect, 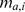, can be calculated using Eq.(2). This equation can be obtained as below by calculating the accumulated drug amount in the medium by adding the removed amount in previous steps to the amount of the drug available in medium at each step.



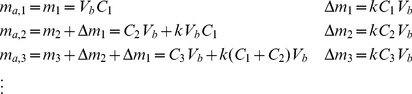
(3)


The adjusted concentration, 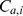 at *i-th* step can be found as below:



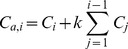
(4)


The proposed method to estimate adjusted concentrations neglects the effect that dilution might have on the diffusion rate. However, for small difference between calculated and measured concentrations, the change in diffusion rate will be insignificant.

### Data Analysis

Results are presented as the mean of six experiments for latanoprost and three experiments for latanoprost free-acid 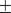 standard deviation. All experiments were performed on different days. To evaluate the significance of the differences between various contact lens materials, *in vitro* corneal models and time points, an analysis of variance (ANOVA) was performed, followed by multiple pair-wise comparisons using the Holm-Sidak test (SigmaPlot, San Jose, California, USA).

## Results

Preliminary studies showed that there was no decay of latanoprost and latanoprost free-acid in the culture medium or buffered solution used in the current research (results not presented), thus enabling the use of the enzyme immuno-assay method to measure drug concentrations in both solutions for up to 48 hours. All the results presented have also been adjusted according to Eq. (4), to take into account the small dilution that may occur as samples are taken out and fresh medium is added.

The uptake analysis showed that 95% of the dissolved latanoprost was taken up by the galyfilcon A and senofilcon A silicone hydrogels and 98% by the balafilcon A (thus approximately 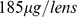) and nearly 25% of the latanoprost solution was taken up into omalfilcon A (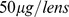).

### Release in the absence of cells

**Figure 1:**
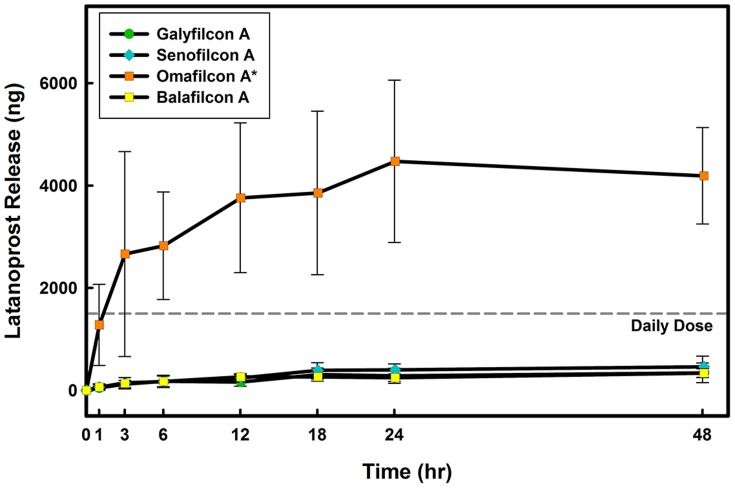
Time course of latanoprost release from four contact lens materials through the no-cells model. Lenses were soaked for 24 hours in drug solution (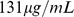) and then overlayed on the model for 24 hours. Aliquots were taken at specific times from the lower compartment and concentrations was measured using EIA. Daily dose line represents the amount of the administered latanoprost for a glaucoma patient [46]. *Significantly different from silicone hydrogel contact lens materials 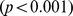. (n = 6 Mean 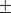 SD).

In the no-cells model, release was first measured in KSFM to allow for comparisons between all *in vitro* models. As shown in Fig. 1, an initial burst in the first 6 hours was observed, followed by saturation, when no more drug was released, despite the available drug in the contact lens material.

**Figure 2:**
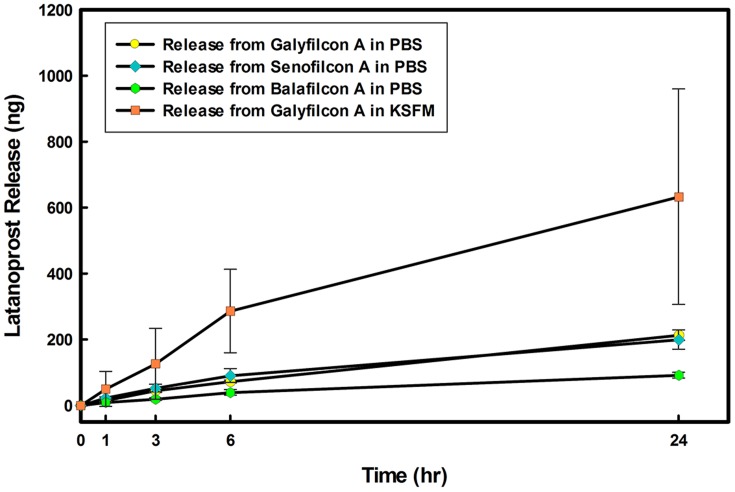
Comparison of latanoprost release from silicone hydrogels in no-cells model. Release from three silicone hydrogel contact lens materials in PBS as well as release from galyfilcon A in KSFM (Keratinocyte Serum Free Medium) is shown. Lenses were soaked for 24 hours in drug solution (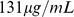) and then overlayed on the model for 24 hours. Aliquots were taken at specific times from the lower compartment and concentrations were measured using EIA. (n = 3 Mean 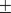 SD).

The effects of the release medium was also assessed with the three silicone hydrogel contact lens materials for 24 hours, where the cell culture medium (KSFM) was substituted with PBS. When compared to KSFM, the latanoprost release decreased significantly in the no-cell model in PBS 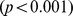, Fig. 2.

### Release in the presence of a monolayer or multilayer model

**Figure 3:**
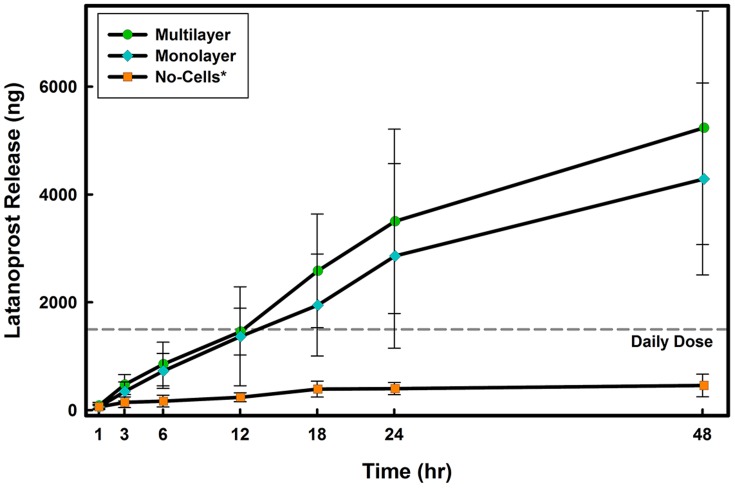
Time course of latanoprost release from senofilcon A in the three *in vitro* models. Lenses were soaked for 24 hours in drug solution (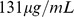) and then overlayed on the model for 24 hours. Aliquots were taken at specific times from the lower compartment and concentrations were measured using EIA. Daily dose line represents the amount of the administered latanoprost for a glaucoma patient [46]. No-Cell Model: Cell culture inserts (PET membrane) without cells, Monolayer Model: PET insert with a monolayer of human corneal epithelial cells, Multilayer Model: PET insert with a multilayer of human corneal epithelial cells (stratified culture). *Significantly different from *in vitro* models with cells 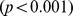. (n = 6 Mean 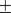 SD).

**Figure 4:**
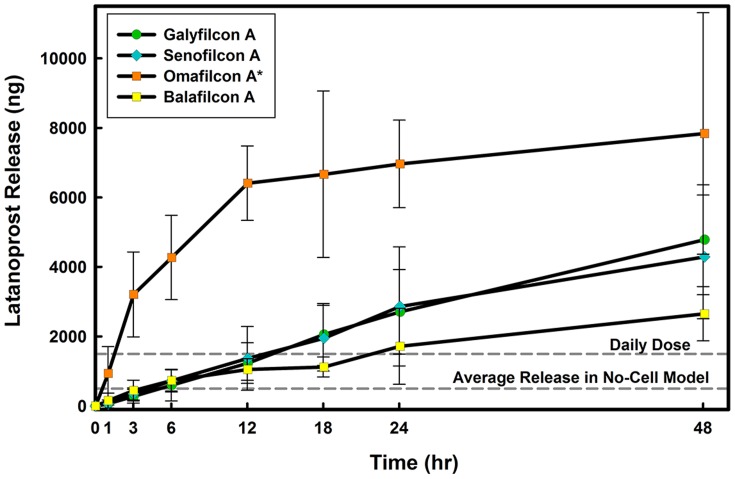
Time course of latanoprost release from four contact lens materials through the monolayer model. Lenses were soaked for 24 hours in drug solution (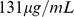) and then overlayed on the model for 24 hours. Aliquots were taken at specific times from the lower compartment and concentrations were measured using EIA. Daily dose line represents the amount of the administered latanoprost for a glaucoma patient [46]. No-Cell Model: Cell culture inserts (PET membrane) without cells, Monolayer Model: PET insert with a monolayer of human corneal epithelial cells, Multilayer Model: PET insert with a multilayer of human corneal epithelial cells (stratified culture). *Significantly different from *in vitro* models with cells 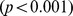. (n = 6 Mean 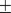 SD).

Performing the contact lens release experiments in the presence of corneal epithelial cells resulted in significant changes. As illustrated in Fig. 3, the amount of latanoprost released from senofilcon A was dependent on the presence of cells in the *in vitro* models; a significantly higher amount of latanoprost was released in the monolayer and multilayer models 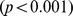 when compared to the no-cell model. Furthermore, while in the no-cell model, no significant difference in release was observed over time, for the monolayer and multilayer models, there was a significant increase in the amount released at 1, 3, 12, 18, 24 and 48 hrs 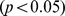. For all contact lens materials studied, in the monolayer and multilayer *in vitro* corneal models, an extended release of drug was observed over time (Fig. 4). The improved release profiles from latanoprost-soaked contact lenses was similar between the monolayer and multilayer models 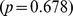.

The release results for all tested commercial contact lenses are summarized in table 2. While the amount of drug released fell within potential therapeutic ranges, only 2% of the amount of the drug sorbed into silicone hydrogel contact lens material was released after 24 hours (Table 2). A significantly higher amount (between 10 to 17% depending on the model used) was released from the high water content hydrogel material, omafilcon A. The high release of latanoprost from omafilcon A (Fig. 4) is in spite of the lower drug uptake, which results in a significantly higher release percentage (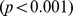, Table 2). Latanoprost release from galyfilcon A and senofilcon A were not significantly different 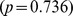 and neither were they different from the release observed with balafilcon A 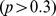.

**Table 2:** Latanoprost Free-Acid Release from Tested Commercial Contact Lenses after 24 Hours.

Contact Lens Material	No-Cells Model		Monolayer Model		Multilayer Model	
	Release	Percentage	Release	Percentage	Release	Percentage
	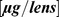	of Release† ( 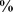 )	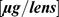	of Release† ( 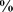 )	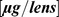	of Release† ( 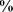 )
Galyfilcon A	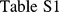	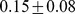	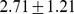 *	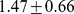 *	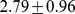 *	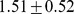 *
Senofilcon A	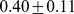	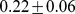	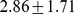 *	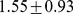 *	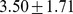 *	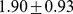 *
Omafilcon A	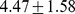 #	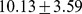 #	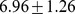 ^#^ *	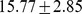 ^#^ *	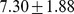 ^#^ *	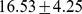 ^#^ *
Balafilcon A	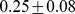	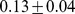	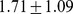 *	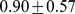 *	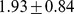 *	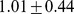 *

n = 6, Mean 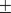 Standard Deviation. Concentration of latanoprost were measured using EIA.

No-Cell Model: Cell culture inserts (PET membrane) without cells, Monolayer Model: PET insert with a monolayer of human corneal epithelial cells, Multilayer Model: PET insert with a multilayer of human corneal epithelial cells (stratified culture).

† The release as a percentage of uptake has been calculated based on the ratio of the released concentration over the sorbed amount.

# Significantly different from other contact lens materials (silicone hydrogel) 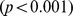.

*Significantly different from the amount released by respective materials in the no-cells model 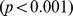.

### Release of Latanoprost Free-Acid

Since in the absence of cells, latanoprost cannot be metabolized to its free-acid form, the release of latanoprost free-acid from contact lens materials was studied to determine if latanoprost free-acid may be used as a substitute to latanoprost in a no-cell model. To allow for a more complete comparison between models and drug forms, release of latanoprost free-acid was also tested with the same *in vitro* models.

**Figure 5:**
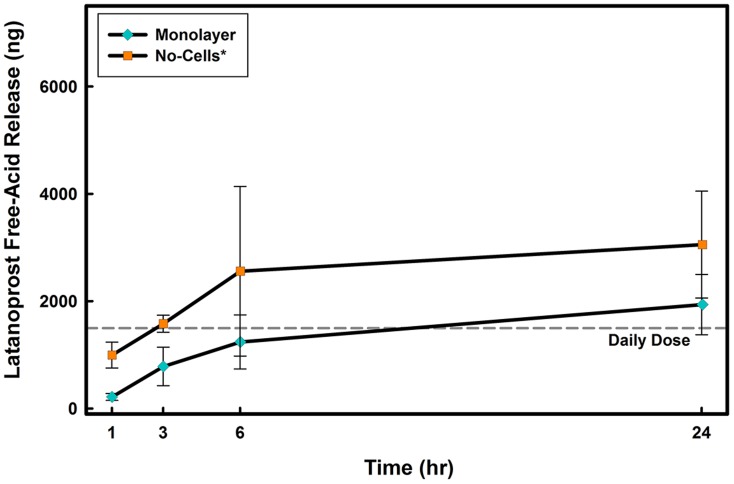
Time course of latanoprost free-acid release from senofilcon A in No-Cell and Monolayer *in vitro* models. Lenses were soaked for 24 hours in drug solution (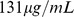) and then overlayed on the model for 24 hours. Aliquots were taken at specific times from the lower compartment and concentrations were measured using EIA. No-Cell Model: Cell culture inserts (PET membrane) without cells, Monolayer Model: PET insert with a monolayer of human corneal epithelial cells. Daily dose line represents the amount of the administered latanoprost for a glaucoma patient [46]. *Significantly different from *in vitro* models with cells 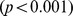. (n = 3 Mean 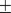 SD).

With latanoprost free-acid, contrary to what was observed with the ester form of the drug, a significantly lower release occurred in the presence of cells when compared to no-cells (Fig. 5). Table 3 presents the release of latanoprost free-acid from tested commercial contact lenses after 24 hours for each of the *in vitro* models. When comparing the amount of drug release at 24 hours in the monolayer model, the latanoprost free-acid results show a significant decrease (approximately 30%) in the amounts of the drug that has been released from galyfilcon A and senofilcon A silicone hydrogels (Table 2).

**Table 3:** Latanoprost Free-Acid Release from Tested Commercial Contact Lenses after 24 Hours.

Contact Lens Material	Release Model 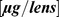
	No-Cells	Monolayer	Multilayer
Galyfilcon A	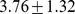	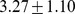	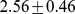
Senofilcon A	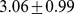	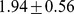	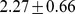
Omafilcon A	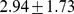	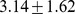	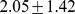
Balafilcon A	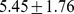 $	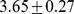 $	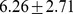 $

n = 3, Mean 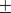 Standard Deviation. Concentration of latanoprost free-acid were measured using EIA.

No-Cell Model: Cell culture inserts (PET membrane) without cells, Monolayer Model: PET insert with a monolayer of human corneal epithelial cells, Multilayer Model: PET insert with a multilayer of human corneal epithelial cells (stratified culture).

$ Significantly different from other lens materials 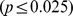.

### The Role of Live Cells

To study the importance of metabolically active cells, which not only provide a physical barrier to drug permeation, but also are able to transfer and metabolize the drug, a set of experiments was designed to compare latanoprost release from the galyfilcon A silicone hydrogel material through a fixed and a live monolayer corneal model. In the fixed monolayer, cells are dead and thus metabolism of the drug cannot occur.

**Figure 6:**
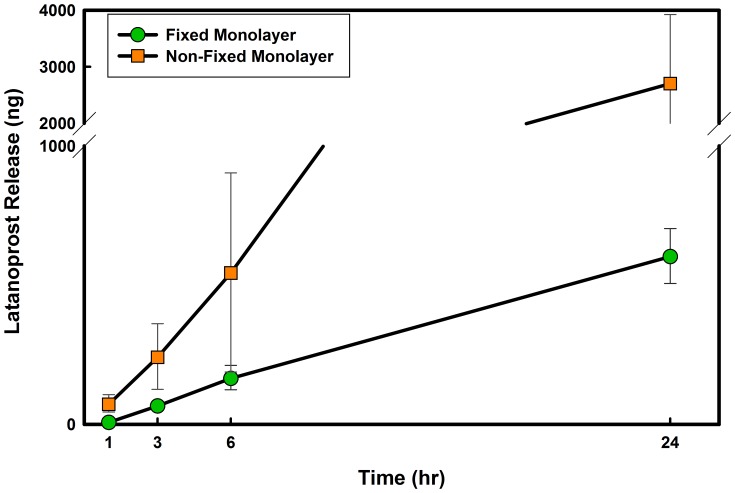
Time course of latanoprost release from galyfilcon A contact lens through live and dead monolayer models. Cells were killed by fixing in Paraformaldehyde. Lenses were soaked for 24 hours in drug solution (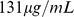) and then overlayed on the model for 24 hours. Release experiments through fixed monolayer were conducted in two separate dates (n = 2, Mean 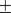 SD). The results were compared to release through monolayer models, (n = 6 Mean 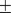 SD).

As shown in Fig. 6, in the presence of fixed (dead) cells, the amount of latanoprost that was released from the soaked galyfilcon A lens and diffused through the monolayer was lower in the presence of paraformaldehyde-fixed cells when compared to metabolically-active cells. These results clearly highlight the importance of the metabolism and transportation in *in vitro* model of drug releasing materials.

## Discussion

This study was undertaken to determine the impact of the presence of cells in *in vitro* models of drug releasing materials. The cells used in these experiments, HPV-immortalized corneal epithelial cells have been used previously by Griffith *et. al*. for corneal constructs and have been shown to exhibit key physiological functions and biochemical marker expression of corneal epithelial cells [38].

Our initial release experiments with drug soaked contact lens material in the absence of cells provided results similar to many others, ([11], [12], [18], [20], [22]) showing a mechanism of a first order release. The limited amount of drug that was released in our fixed volume model is likely the result of the high partition ratios of the latanoprost between the contact lens material and the aqueous solutions. Furthermore, our results from the no-cells model suggest that latanoprost has a lower affinity toward PBS compared to KSFM. The better solubility of latanoprost in KSFM compared to PBS is likely due to the difference in composition, such as the presence of growth factors and other ionic compounds in the culture medium which are absent in the buffered saline solution. While, in our experiments, the nature of the medium was found to have a statistically significant impact on release in the no-cell model, the actual improvement in drug release is actually insignificant when compared to the *in vitro* models with cells.

Significantly higher drug release and diffusion were observed in the presence of cells. Due to their hydrophobicity, ester prostaglandin analogues, such as the latanoprost prodrug, have a greater chance of diffusion through the hydrophobic corneal epithelium [43]. Furthermore, metabolism will also play a role in the presence of live (metabolically active) cells, since the latanoprost prodrug is expected to be metabolized by cells [27], [29], [30], [46] before diffusion through the cell layer. The metabolized product, the latanoprost free-acid, has a smaller partition ratio and is more water soluble when compared to latanoprost. Therefore, the presence of cells will improve the drug diffusion rate. A layer of cells will also improve drug release from the contact lens material by maintaining the concentration gradient between the lens and the solution above the cells through metabolism of the latanoprost.

A recent study showed that the nonmetabolized (ester) form of latanoprost contributed to only 4% of the total drug diffused through an *in vitro* corneal model and that no detectable amount of ester form of the latanoprost was observed in an *ex vivo* model [26]. We may thus assume that the majority of the diffused drug through the *in vitro* corneal models with cells is the free-acid form.

Different latanoprost release profiles were observed among the hydrogel contact lens materials tested. Compared to the silicone hydrogel materials, the high release of latanoprost from omafilcon A in spite of the lower drug uptake may be explained by the low affinity of the latanoprost (an hydrophobic compound) toward the omafilcon A contact lens material, which is a high water content hydrogel. The large partition ratio results in a low uptake by this material when soaking in aqueous solution of hydrophobic drugs, as well as relatively fast release rates in the release solution.

Using latanoprost esterified form, i.e. the active drug compound, latanoprost free-acid, affected results in all models. Higher water solubility of the latanoprost free-acid led to higher amounts of drug being released from the silicone hydrogel lens materials in the no-cell model. As a more polar molecule, latanoprost free-acid has a lower partition ratio between the hydrophobic silicone hydrogel contact lens materials and the aqueous solution when compared to latanoprost. While higher amounts of latanoprost free-acid were released in the no-cell model, lower releases were observed in the presence of cells. With latanoprost free-acid, epithelial cells now act as a barrier against the diffusion of the latanoprost free-acid, and hence limit the diffusion of the hydrophilic drug.

As one compares the latanoprost and latanoprost free-acid release results, it becomes evident that similar drug release profiles cannot be obtained by replacing the prodrug with the drug, even in the no-cell model. Not only are the amounts released significantly different by an order of magnitude, but while all silicone hydrogel materials released similar amounts of latanoprost, balafilcon A released significantly more latanoprost free-acid compared to the other two silicone hydrogels. The balafilcon A/latanoprost free-acid results are likely due to the fact that balafilcon A material has an overall net negative charge due to the incorporation of some acidic material components [46] and its surface charge increases the hydrodynamic attributes of the material [47], therefore increasing the role of adsorption of the hydrophilic drug on the surface of the contact lens during the uptake process and its subsequent release in solution. Nevertheless, taken together, our latanoprost free-acid results highlight the relevance of using *in vitro* models with cells when studying release of a prodrug that requires to be metabolized before diffusion through the tissue to the site of treatment.

Due to the lack of previous *in vitro* studies on prostaglandin analogues, our results can only be compared to the release of drugs from the contact lens materials with similar size and hydrophobicity. Previous *in vivo* studies have shown a prolonged release of relatively hydrophobic drugs such as ketotifen [21] and lomefloxacin [23], however such release profiles could not be replicated *in vitro* using a fixed volume release model [12], [22]. The extended release of latanoprost observed in the monolayer and multilayer *in vitro* models correlates well with the extended release profiles of the hydrophobic drugs observed *in vivo* [21], [23]. The release results of latanoprost in the no-cells model is also comparable to the release results of hydrophobic compounds in fixed volume solution [12], [22]. The significant role of cell metabolism and transport was further demonstrated using fixed (metabolically inactive) cells. Taken together, our results suggest that the absence of cells in *in vitro* models of drug release likely contributes to the contradiction between these *in vitro* and *in vivo* studies [12], [21]–[23].

## Conclusion

Poor release results from commercially available contact lens materials soaked in hydrophobic compounds such as latanoprost have been obtained with fixed volume release models similar to the no-cells model used here. However, we have demonstrated, using drug-soaked silicone hydrogel materials, that the amount of drug diffusing through an *in vitro* corneal model is in the order of 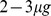 over a period of 24 hours, which is comparable to the 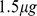 of drug in every drop of the commercial latanoprost. Our results emphasize the importance of the presence of cells when characterizing the release of drug-delivery materials and demonstrate how experimental *in vitro* models have a significant impact on the outcomes of testing ophthalmic drug delivery materials. Our *in vitro* study suggests that silicone hydrogels have the potential to deliver latanoprost effectively over an extended period of time.
